# Patient Experiences of Swallowing Exercises After Head and Neck Cancer: A Qualitative Study Examining Barriers and Facilitators Using Behaviour Change Theory

**DOI:** 10.1007/s00455-017-9799-x

**Published:** 2017-04-19

**Authors:** Roganie Govender, Caroline E. Wood, Stuart A. Taylor, Christina H. Smith, Helen Barratt, Benjamin Gardner

**Affiliations:** 1 Research Department of Behavioural Science & Health, University College London, London, UK; 2 University College London Hospital, Head and Neck Cancer Centre, London, UK; 3 UCL Centre for Behaviour Change, Research Department of Clinical, Educational and Health Psychology, University College London, London, UK; 4 Centre for Medical Imaging, University College London, London, UK; 5 Division of Psychology & Language Sciences, University College London, London, UK; 6 Department of Applied Health Research, University College London, London, UK; 7 Department of Psychology, Institute of Psychiatry, Psychology and Neuroscience (IoPPN), Kings College London, London, UK; 8 Department of Epidemiology & Public Health, University College London, London, UK

**Keywords:** Dysphagia, Swallowing exercises, Adherence, Behaviour change, Qualitative interviews, Content analysis, Theory-based interventions

## Abstract

Poor patient adherence to swallowing exercises is commonly reported in the dysphagia literature on patients treated for head and neck cancer. Establishing the effectiveness of exercise interventions for this population may be undermined by patient non-adherence. The purpose of this study was to explore the barriers and facilitators to exercise adherence from a patient perspective, and to determine the best strategies to reduce the barriers and enhance the facilitators. In-depth interviews were conducted on thirteen patients. We used a behaviour change framework and model [Theoretical domains framework and COM-B (Capability–opportunity–motivation-behaviour) model] to inform our interview schedule and structure our results, using a content analysis approach. The most frequent barrier identified was *psychological capability*. This was highlighted by patient reports of not clearly understanding reasons for the exercises, forgetting to do the exercises and not having a system to keep track. Other barriers included feeling overwhelmed by information at a difficult time (lack of *automatic motivation*) and pain and fatigue (lack of *physical capability*). Main facilitators included having *social support* from family and friends, the desire to prevent negative consequences such as long-term tube feeding (*reflective motivation*), having the skills to do the exercises (*physical capability*), having a routine or trigger and receiving feedback on the outcome of doing exercises (*automatic motivation*). Linking these findings back to the theoretical model allows for a more systematic selection of theory-based strategies that may enhance the design of future swallowing exercise interventions for patients with head and neck cancer.

**Electronic supplementary material:**

The online version of this article (doi:10.1007/s00455-017-9799-x) contains supplementary material, which is available to authorized users.

## Background

Rehabilitation of swallowing function after treatment for head and neck cancer (HNC) requires patients to adhere to swallowing exercise interventions. However, adherence is generally reported to be poor [1–3]. Studies aiming to establish the effectiveness of exercise interventions for this population often neglect this aspect [4, 5], and may consequently portray effective interventions as ineffective. Improving patient adherence is one way of optimizing interventions prior to evaluation, although the most effective methods to improve adherence remain unclear. Techniques to increase adherence are likely to be more effective if they are informed by in-depth exploration of patients’ experiences of their swallowing exercises, probing both barriers and facilitators to adherence.

Patients presenting with HNC undergo a protracted journey from diagnosis through to treatment, rehabilitation and long-term follow-up with up to two-thirds experiencing dysphagia before treatment [6]. The swallowing sequelae of surgical and non-surgical treatments are well documented and often predictable [7–9]. Clinicians have a unique opportunity to intervene early in the patient pathway [10, 11], and establish swallowing exercise programmes that may potentially enhance post-treatment outcomes [3, 12–18]. In a retrospective study of prophylactic swallowing exercises, patients who adhered most to their exercises were more likely to be tolerating a more regular diet one month post-treatment than non-adherers. Similarly, dependency on a gastrostomy tube was reported to be higher in patients who were non-adherent to exercises [19].

Some work has been undertaken to understand underlying reasons for non-adherence to swallowing exercises. In a telephone survey, Shinn et al. [1] reported that rates of complete non-adherence (did not do the exercises at all) were high (55%) with a further 36% reporting only partial adherence. Common reasons given by patients for non-adherence were as follows: not having a swallowing problem at the time and lack of understanding of the need for exercises, finding exercises difficult, forgetting to do them, being too busy, experiencing pain, nausea and fatigue.

A more recent study [20] examined adherence to a 12-week preventative programme and investigated whether demographic (age, gender), clinical (tumour site and stage, and treatment modality) and health-related quality of life (HRQOL) were associated with exercise performance. The percentage of patients who adhered to the programme at least once daily for the duration of the study was 70% at 6 weeks, dropping to 38% at week 12. The addition of chemotherapy to the radiotherapy regime was the only significant factor associated with poorer exercise performance. This concurs with the findings of Shinn et al. [1] who reported that pain, nausea and fatigue in patients having chemo-radiation were barriers.

Previous studies have used mainly *deductive* methods to identify reasons for non-adherence, based on commonly endorsed researcher-generated ideas. *Inductive* methods using in-depth interviews that seek to spontaneously elicit the reasons, belief systems, attitudes and underlying values from patients provide a rich source of context-relevant information from a patient perspective. This may yield important additional barriers to exercise performance and adherence that may be highly relevant, but possibly less intuitive to the researcher. As this approach elicits the overall experience of patients, we may also learn which factors facilitate doing the exercises. Optimizing facilitators is another way of potentially improving the design of interventions. To our knowledge, no study has explored the problem of poor patient adherence to swallowing exercises amongst the HNC population using in-depth patient interviews guided by a theoretical framework. Theoretical frameworks of behaviour change, rooted in behavioural science, offer useful tools for exploring and organizing reasons for adherent/non-adherent behaviours. It has been suggested that interventions aimed at modifying behaviour are more likely to be successful if based upon theory. Theory allows researchers to be more systematic and explicit in investigating mechanisms of change [21], and has been demonstrated to have useful application in other aspects of speech and language therapy practice requiring behaviour change [22]. In using theory, we may accumulate knowledge incrementally, building on existing scientific knowledge.

This study is part of a larger project aimed at developing an optimized swallowing intervention package for patients with HNC. The purpose of the present study is to identify key factors (those most commonly reported by patients as being important to them) that may inform the design of a new intervention. Using behaviour change theory, the identification of barriers and facilitators (things that hinder or promote adherence) to performing swallowing exercises represents the first step in a behavioural analysis [23]. Categorizing findings according to a behavioural model could help identify the most useful strategies to minimize the barriers and enhance the facilitators.

The study received full ethical approval from a National Health Service (NHS) ethics committee (14/LO1152).

## Methods

### Design

We used face-to-face semi-structured interviews to explore and understand the personal meanings, experiences and issues pertinent to individuals in the context of their swallowing rehabilitation. We developed a topic guide that allowed participants the flexibility and freedom to narrate their experience of eating and drinking and swallowing rehabilitation over the course of their cancer treatment. Questions and probes were used to ensure that topics of interest were covered in adequate depth.

### Theoretical Framework

We have drawn upon theoretical models from behavioural science namely, The theoretical domains framework (TDF) [24, 25] and the COM-B (Capability, opportunity, motivation behaviour) model [26] to guide understanding of patients’ exercise adherence behaviours and experience of swallowing rehabilitation. The framework and model were used both in developing the interview schedule as well as informing the content analysis approach used.

**Fig. 1:**
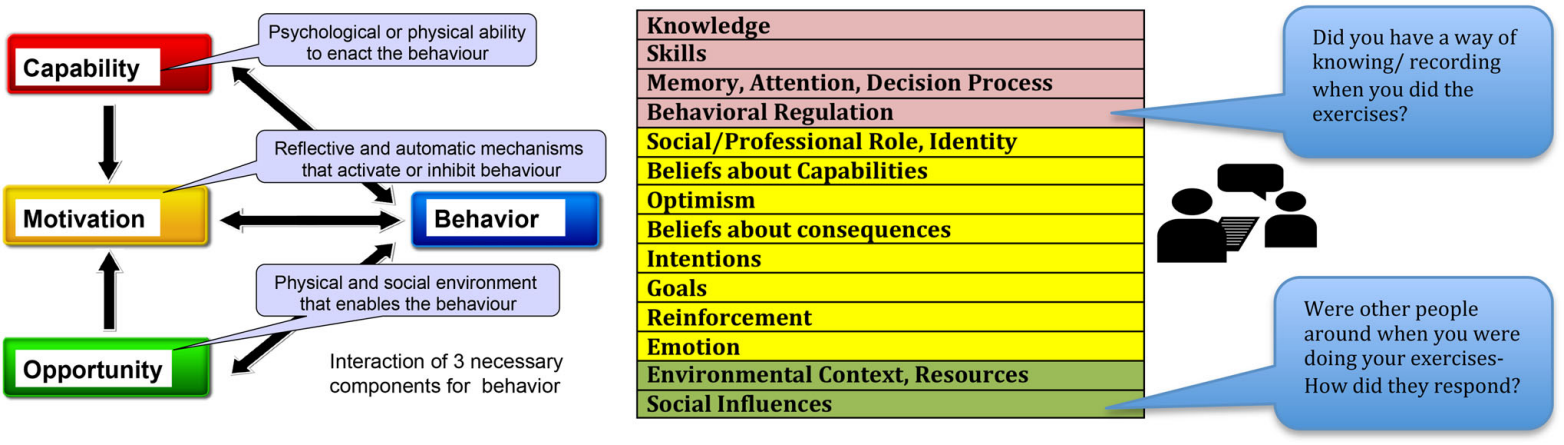
The COM-B model and the 14 associated domains of the Theoretical Domains Framework. *Adapted from [23]

A topic guide was developed using the TDF [24, 25] as a basis for prompt questions. The TDF consists of a comprehensive set of 14 domains into which all determinants of adherence to/implementation of a behaviour can be organized: *knowledge, cognitive and interpersonal skills, memory and decision processes, behavioural regulation, social influences, social professional role and identity, beliefs about capabilities, optimism, intentions, goals, beliefs about consequences, re*-*inforcement and emotion.* The TDF can be mapped onto the over-arching COM-B model [26] which posits that three key components are necessary for any behaviour—capability, opportunity and motivation. For a behaviour to occur, an individual must have both the *physical* and *psychological capability* to perform the behaviour in terms of the mental and physical skills, knowledge, strength and stamina. The *physical* and *social environment* for example having the time, physical space, resources, support from others affords *Opportunity*. *Motivation* may be described as *reflective* where an individual is consciously involved in planning. This is based on his/her evaluations of whether something is good or bad to do, on whether it meets their goals, and their self-belief that they can perform a behaviour in spite of obstacles. *Automatic motivation* on the other hand is driven by impulses, emotional reaction or reflexive processes such as a trigger to perform a behaviour that has become habitual. Performing daily swallowing exercises is the primary *target behaviour* in most swallowing interventions, and is therefore the main subject of enquiry in this qualitative study. Figure 1 depicts how the topic guide (available as supplementary information) was developed using the theoretical framework to ensure comprehensive coverage of the key components that drive behaviour.

The topics included aspects such as knowledge of swallow exercises, ease of carrying out exercises, beliefs about exercises, feelings and emotions, and support for doing exercises. The interview opened with a general and broad question: *Can you tell me how you got on with eating and drinking at the time of your treatment?* Follow-up questions and probes were introduced as part of the narrative flow rather than as individual discrete questions. Patients were encouraged to speak freely about their experiences with swallowing rehabilitation.

### Participants and Sampling

All participants (patients) were recruited via clinicians working in the head and neck cancer centre at a UK metropolitan teaching hospital. Clinicians were asked to identify patients who had received treatment for advanced head and neck cancers. Patients who were between 3 and 18 months post-treatment were sought, as they were deemed sufficiently beyond the acute phase of recovery but still likely to reliably recall their experiences. Patients were required to have undergone swallowing rehabilitation including a minimum of three swallowing exercise consultations with a speech and language therapist (SLT).

The sample size was determined using the ‘ten plus three’ rule for data saturation [27]. An initial target of ten patients was set, with a view to achieving a point where three consecutive interviews could be undertaken without new themes emerging. Importantly, it was necessary to include as much diversity in the sample to ensure a good representation of socio-demographic factors. For this reason, midway through the recruitment, selected characteristics of participants were examined (age, gender, treatment modality and swallow function). Attempts were then made to purposively recruit participants with characteristics that were lacking from the existing sample, in order to ensure a broad range of experiences. Table 1 shows a summary of participant characteristics.

**Table 1:** Summary characteristics of sample

Characteristic	Sample (*n* = 13)
Age *n* (mean)	
60 years and over	4 (63)
Under 60 years	9 (50)
Gender	
Male	9
Female	4
Treatment	
Surgery and chemo-radiation therapy	4
Chemo-radiation therapy	5
Surgery and chemotherapy	1
Radiation therapy	3
Swallowing status at time of interview:	
Performance status scale (PSS)	
50 and over (soft diet and better)	9
Under 50 (liquids, puree, NBM)	4
Time since treatment	
3–6 months	6
6–12 months	3
12–18 months	3
Beyond 18 months	1
Gastrostomy tube during treatment	
Yes	11
No	2
Still in situ at time of interview	6
Marital status	
Married/co-habiting	8
Single/separated	5
Occupational category	
Graduate professional	2
Manager/director	2
Caring/leisure/service industry	2
Professional/technical	1
Skilled trade	2
Admin/secretarial	1
Retired/not employed	3

### Procedure

All patients provided written consent. All interviews were conducted by the lead researcher (RG), who is also a SLT clinician, previously unknown to the patients. Interviews lasted 40 min on average. A few minutes were spent before each interview completing basic biographic data and allowing time for questions about the study. This afforded patients time to relax into the environment and an opportunity for the interviewer to establish rapport. Interviews were digitally recorded and professionally transcribed verbatim. To ensure full anonymity on the recording, participants chose a pseudonym for themselves at the interview outset. Transcripts were imported into NVivo 10 (QSR International) to organize analysis.

### Analysis

The analysis was undertaken by the researcher (RG) in three stages, drawing upon the content analysis method. Content analysis is well suited to research questions that use context-relevant information generated from the interviews to re-populate pre-specified theoretical constructs [27]. *Familiarity with data and initial coding* involved listening to the recording, making notes and assigning initial codes to sections of text. *Refinement of codes and development of a codebook* were then undertaken by the researcher (RG). Codes were grouped into clusters that reflected broader themes and duplicate or redundant labels were removed. This was a recursive process that often required reading and re-reading content coded with the same label across interviews to ensure that it was an accurate depiction of the concept. Once a satisfactory coding system was achieved, codes were matched to the domains of the TDF. A working codebook was developed by the lead researcher/first coder (RG) to allow verification by a second coder (CW), with expertise in both qualitative analysis and the use of the TDF. *Verification of coding and peer debrief* was undertaken by the second coder (CW) using the codebook to independently code three randomly selected transcripts. This served to examine reliability and improve validity thereby adding rigour to the analysis [28]. The peer debrief focused on three aspects which included *comprehensiveness of the codebook* (all relevant content could be attributed a code label), *degree of agreement for the presence of codes* (percentage agreement by both coders for the presence of codes in each transcript) and *degree of uncertainty* (any uncertainty with regard to description of code labels, TDF domain to which code assigned, need for new codes). Agreement on the presence of codes was above 90% for each of the transcripts. Uncertainties were resolved through discussion. Following this process, the first coder (RG) undertook a final reading of the transcripts to ensure that all content was appropriately coded, particularly where changes were made following the peer debrief. At the final step, coded material was re-aligned to the theoretical model to determine which variables may need to be targeted to bring about change.

## Results

A total of 13 patients were interviewed to achieve data saturation. As indicated in Table 1, a range of patient characteristics was achieved. Tables 2 and 3 illustrate the *key* barriers and facilitators identified in greater than 50% of interview transcripts, and the corresponding mapping onto the relevant COM-B component.

**Table 2:** Key barriers to swallowing exercises

Key barriers	COM-B	Examples
Inadequate knowledge of how treatment will affect *own* swallowing.	Psychological capability	The doctor scribbled down a few symptoms that I would suffer after the radiotherapy, one of which was sore throat and one of which was maybe problems with the swallowing, or something along these lines (P12) They told me I will need a feeding tube, I will have a feeding tube. Even if I don’t use it they are going to give me a feeding tube, because, I don’t know, for example, nine out of ten patients, at some point during treatment, won’t be able to take food. So I will definitely need one (P13)
Inadequate understanding of why exercises given pre-treatment	Psychological capability	I understand someone sitting there explaining to me that you will need to do these exercises to help you swallow, but I don’t think the emphasis was how important they were, for me. I don’t think I actually took that on board (P3) I was given some leaflets on swallowing exercises and told that I would probably get a dry mouth and that would cause problems with swallowing (P11)
Forgetting to do exercises, no system of keeping track	Psychological capability	It was a bit random; I would just do it when I remembered, some of the time (P1) I think what I’m remembering and what I’m saying is because there wasn’t a discipline around it, sometimes they slipped a bit (P9)
Overwhelmed by information at a difficult time (emotion)	Automatic motivation	Loads and loads of stuff was happening that was unfamiliar and a bit scary, and so, you know, I, sort of, felt a bit bombarded with stuff (P1) There was a lot to take in during that period. This is something else to take in as well, necessary but not life… This isn’t going to save your life; this is going to make it better afterwards. Very important. But as a patient, when you are faced with a life-threatening situation, I think that wouldn’t be a priority and you’d want to push that away for now (P5)
Pain and fatigue	Physical capability	I tried to do some of the exercises some of the days. And some of the exercises I just couldn’t do because of the pain I was actually experiencing that particular day (P3) When I got tired from the chemotherapy and so forth, I think I let it all, kind of, go a bit (P2)

**Table 3:** Key facilitators to swallowing exercises

Key facilitators	COM-B	Examples
Support from clinician and family	Social opportunity	So I think it was before and it was during, right up until I could eat again, I was constantly getting advice and help (P13) I started doing exercises, the throat exercises and eventually… it took some time, but I was told by my family as well that don’t give up. Because at that time I was just about to be a grandfather as well and that also gave me the strength (P10)
Desire to prevent negative consequences from treatment	Reflective motivation	But I don’t know, I just knew I had to eat, you know. And my object was not to use that… what do you call it? The tube they stick in you. And I managed it. I didn’t really use the tube (P6) I thought, well, if you don’t use muscles, they, sort of, stop working, don’t they? I’ve seen it with people with broken legs. If they don’t use them the muscles wither. And so I thought if that’s just going to happen to my throat, I don’t want that happening (P7)
Knowing how to do the exercises (skills)	Physical capability	The exercises themselves were pretty simple exercises using the tongue and biting, protruding the tongue between your lips and holding onto the tongue and trying to swallow, to do with breathing and holding your breath while you swallow. They were pretty simple tasks (P3) After the first week you could do them whatever they were, even just go through them through your head. Yes. It would be like going to the gym and doing ten different classes and you know all the steps. It’s the very same. It’s familiarity, isn’t it? (P4)
Having a routine and/or having a trigger to do the exercises (behavioural regulation)	Psychological capability	My exercises at the beginning, I’d actually write them on the chart. But what I used to do is I’d put them on… I’ve got an iPhone (P3) I had a form from the team and I used to mark down how many - on a Monday, four times, I’d mark it off four times, Tuesday four times, all the way up to Thursday. And I didn’t do them on Friday. It was a Friday morning. I had it marked out on the chart and you give the chart when you come in for the exercises, she’d have a look at it. She’d say, ‘Yes, you are doing well’ (P4)
Receiving feedback on outcome (re-inforcement)	Automatic motivation	You are achieving something every time. And they tell you, yes, you are doing very good and they tell you it’s open so many centimetres today, and then they’d compare it from last week. They’d have it written down (P4) I took a short drink, the energy drink, and I started drinking it and he was… my son and my daughter as well were so pleasantly surprised. They were, sort of, overcome with joy. So there was a joy that I could drink at least (P10)

### Capability

*Psychological capability* was the primary component identified as a barrier to patients’ adherence to swallowing exercises. This encompasses the psychological skills including the mental stamina and processing of knowledge and information [26]. Patients recounted being given information but not necessarily relating this to why they might need to do their swallowing exercises. In addition to feeling that the pre-treatment exercises were just a precaution, some patients did not give much credence to the exercises themselves. This is exemplified by the following patient quote:

They just said to me, ‘Do that three times a day, whatever, in the morning and night.’ [*talking about the exercises he was given*] I thought what’s it going to do?… What they told me, for the amount of times to do it, I thought it was just someone wrote it 100 years ago and it’s still the same rules (P7, male).

Patients also talked about the number of competing priorities during treatment and the cognitive burden of trying to do many different things just to get through treatment. A few patients mentioned the difficulty in knowing what to prioritise.

I met with the speech and language people early on; I met before I started treatment. And they talk about do[*ing*] your exercises through treatment as well. But it becomes a matter of priorities when you are in treatment and it’s really rough, and unfortunately that one just gets pushed… well, for me it did, it just gets pushed to the back of the queues, trying to get the mucus out of my system, yes, trying to stay hydrated, trying to keep the pain under control. And when it was really bad, speech and language is the furthest thing from your mind (P8, male).

*Physical Capability* was also a barrier for patients during treatment when side-effects such as pain, nausea and the presence of sticky secretions in the mouth took precedence and patients looked for an easier solution to obtaining their nutritional requirements.

Certainly with a PEG in you needn’t swallow at all. You’ve got to keep your mouth moist but that’s all you need to do (P12, male).

Some aspects of *Physical and Psychological Capability* were also identified as potential facilitators. Generally patients felt that the exercises were simple and easy to perform once they learned to do them and were confident they were doing them correctly. Patients who incorporated a method for self-regulation, such as marking off the exercises on a chart or using a smart-phone to keep track reported these to be helpful strategies.

### Opportunity

*Physical opportunity* factors, which encompass the environmental context and resources [23], generally did not feature as a commonly reported barrier. However, a few patients with children felt that they were less keen to do the exercises with the children around.

During most of my treatment I spent a lot of my time thinking about the boys rather than myself, so how would things… what I could hide from them or what I could make unscary for them, what I could tell them (P2, female).

Some patients felt that most of the exercises could be done anywhere:

Because the exercises generally were over maybe two or three times a day, different exercises. It’s something you could do in the car when you were driving, or whatever, they didn’t have to be in situ (P5, male).

While others felt that they needed a space or preferred privacy for some of the exercises.

I remember lying on the floor in the landing, I remember lying on the floor in the bedroom trying to fit them all in, There was that sense of needing to have a space to do some of those [*reference to Shaker, head lift exercise*]. Yes. I think some of the noisy ones I would sometimes do when I walked the dog on the heath (P9, female).

The provision of resources relates to *physical opportunity* to perform the exercises. Some patients felt that the method of information provision could be improved and that pictures might have enabled a better understanding of the exercises.

I don’t know. Maybe pictures with diagrams or something to show what part of your tongue you should be tensing up, like more emphasis on when you are swallowing, because you weren’t sure really if it was the front of your tongue or the back of your tongue, sort of, to be pushing up (P11, female).

Additionally, one patient in particular highlighted the need for re-structuring in the approach taken as many people are resistant to being *told* what to do.

Prescriptive is the word I was looking for before. I felt that the people I was dealing with generally were kind of prescriptive. Do you know what I mean by that? (PI, male).

*Social opportunity* in the form of social support from others (family members, other patients and clinical staff) was a strong positive influence in facilitating adherence to the exercises. Patients who had someone offering encouragement tended to adhere better to their exercises. A few patients reported that their children would often get involved in overseeing their exercises.

My daughter, who is seven, felt the need to copy me when I was doing my floor exercises, which is a great tonic because it felt like you were making a game of it, which is quite nice. And that’s something to encourage people, if they do have younger children, because it takes that onerous edge to it away, I think. She took over the situation and became my speech therapist, physiotherapist and nurse all rolled into one, bless her cotton socks. In all seriousness, throughout the whole journey of last year she was an enormous encouragement to me without saying a word, to make sure that I could get back to somewhere, near to where I was. That’s what makes life worth living really, the children (P5, male).

### Motivation

*Reflective motivation* involves the psychological processes that drive behaviours that serve a goal deemed a priority by the individual. It includes conscious planning and weighing up whether performing a particular behaviour is beneficial to the end goal [23]. Additionally, the individual’s belief (self-efficacy) that they can overcome obstacles to performing the behaviour in order to attain their goals is an important element of motivation.

I knew if I did not eat I would not have the strength to fight the illness. So I said, for myself, for my family’s sake and everyone’s sake I have to fight (P10, male).

It’s your own tenacity to get better (P5, male).

For some patients motivation was impeded by physical and psychological capability: the feeling that there was too much to do, or the uncertainty about the relevance of the exercises to their own unique circumstances, particularly if they were given prophylactic exercises.

I don’t know how long the full set is. If you are doing three reps it’s… it’s hours a day, particularly when you’ve got the emphysema exercises bolted in. And that’s quite hard to achieve (P12, male).

It’s completely impossible to envisage what your throat and mouth and tongue might feel like if you are a healthy person. So doing things like holding your tongue and trying to swallow [*masako*—*tongue base exercise*], you do it, but you don’t know why, and it feels sort of slightly kind of worrying (P2, female).

*Automatic Motivation* is less conscious and more reflexive, driven by emotional states, impulses and context triggers. This aspect of the COM-B model is represented by the theoretical constructs of *Reinforcement* and *Emotion* on the TDF [23]. Individuals described feeling rewarded by small improvements in their swallowing which motivated them to do their exercises in the hope that they could achieve more. This included receiving positive feedback about the outcome of doing their exercises (for example increased mouth opening, seeing with biofeedback that they could reduce aspiration) or experiencing an improvement in function such as the ability to drink something after a long period of being unable to.

One of the nicest things is when you are…. And you can’t drink water and you rely on all your fluids through the PEG, and you get to the point where you can just get a sip of water down, and you get that sip of water down and you keep working on that sip of water. But you get points where you are thirsty and you want to drink like a normal person. Getting to the point where you can drink is a real breakthrough. That makes a massive difference to just your overall feeling and wellbeing, because you stop bunging fluid in here [*pointing to PEG tube*]… And you can, you know, have two or three mouthfuls without stopping (P8, male).

The results presented above suggest that there is potential to optimize all three key components of behaviour to improve swallowing exercise interventions for patients after HNC. However, *capability* seems to require the greatest shift in order to bring about a change in patients’ exercise adherence behaviour.

## Discussion

This study described a theory-based qualitative approach to exploring and categorizing patients’ experiences of their swallowing rehabilitation and reasons for adherence/non-adherence to swallowing exercises. We used an inductive approach to elicit patient experiences and a deductive method to make a “*behavioural diagnosis*” using a theoretical framework [24, 26].

Our results confirmed earlier findings regarding common barriers to swallowing exercise adherence [1]. Additionally, we categorized these findings according to the three key drivers of behaviour which may then inform the selection of appropriate behavioural strategies. Patients indicated that they did not clearly understand the reasons for doing exercises highlighting that *capability* was a key barrier. Interview findings suggest that knowledge and understanding of how swallowing will be affected and why exercises are required may not be sufficiently processed by patients, particularly if they are given exercises at pre-treatment stage. The importance of information provision for this patient population has received considerable research attention [29–34]. On the one hand, clinicians aim to provide all the necessary information, yet researchers report that patients may not *take in* all this information. More information is therefore not necessarily the solution to the barrier of lack of knowledge and understanding. Patients in this study were able to reflect on their own pre-treatment counselling and reported that it was important to find a balance between helping people *understand* how and why their eating and drinking might be affected and not “over-scaring” them. Patients themselves highlighted that while a great deal of information is provided verbally and in the form of leaflets, they dismiss much of it as they do not consider it personally relevant to them. Many patients reported feeling overwhelmed and therefore chose to filter information they received. Consequently, they dismissed the exercises as being a general precaution, believing that it was not relevant to them. This was particularly the case if they were able to eat and drink adequately at the time.

Some patients preferred not to know about negative consequences of treatment, as they felt that this added to their anxiety. One patient in particular felt that the approach was too prescriptive. These results suggest that there is scope to improve delivery of information about treatment and its impact on function so that patients clearly understand the relevance to them. At pre-treatment, some patients were keen to learn how they may best help themselves over the course of their treatment. It may therefore be useful to explore ways of creating and capitalizing on a *teachable moment* that may be co-created by the clinician–patient interaction [35].

As expected, participants reported varying *physical capability* to perform the exercises. Based on the higher numbers of patients who reported that pain was a barrier to doing their exercises, greater effort may be needed to minimize this problem. Other researchers have likewise alluded to the fact that increased and uncontrolled pain and toxicity from treatment reduce patient adherence and maintenance of swallowing exercises [16, 20]. A study by Starmer et al. [36] reported improved pain control, and swallowing function in 23 patients treated with gabapentin in the first week of radiotherapy compared to 23 matched controls who did not receive gabapentin. Further work is required to assess the value of administering early pain control for this group of patients in relation to maintenance of swallowing and swallowing exercises.

Patients who were able to master the exercises before treatment and developed a system to build the exercises into their daily routine were better at maintaining them throughout the treatment. It seems plausible to relate this finding to previous work in behavioural science that has highlighted that forming *habits*, that is ingrained automatic routines initiated by environmental cues, may be important to maintaining long-term behaviour [37, 38]. Habits form through context-dependent repetition [39], and while initially effortful becomes easier if the action is repeated with sufficient consistency in the same position within one’s routine [40, 41]. This is particularly crucial in the early stages in order to facilitate habit formation [42]. The advantage of exercises becoming habitual is that they are more likely to be maintained over time, as they become less reliant on motivation and other cognitive processes such as conscious memory [39]. These insights could be usefully applied in the design of pre-treatment swallowing exercise interventions.

*Physical opportunity* (*environmental and resources*) did not feature prominently as a barrier. This may be explained by the fact that most of the swallowing exercises do not require many resources once they are mastered, and for the most part can be done anywhere. Patients who reported time and space concerns also seemed to reflect on whether they used this as an “excuse” to justify to themselves why they may not be doing their exercises. *Social opportunity,* however, seemed a strong facilitator in that patients who had support from a friend or family member offering encouragement were more likely to have kept up the exercises. Regular appointments and support from the SLT to keep up the programme also appeared to be an important facilitator. In our earlier literature review study, we identified *social support* as one of the main behaviour change techniques in successful swallowing exercise interventions [5].

*Reflective motivation* is strongly linked to psychological capability [23]. Individuals were unlikely to set a goal such as *being able to eat after treatment* if they did not perceive this as a potential problem that will affect them. Most individuals talked about wanting to avoid a feeding tube, hoping to maintain the ability to eat and drink by mouth throughout the treatment. For patients who recognized that swallowing function might be impaired, a desire to prevent negative consequences such as reliance on a gastrostomy tube was identified as an important facilitator for initiating swallowing exercises. Other patients indicated that despite feeling motivated initially, the ability to follow through with exercises during a challenging course of treatment was often eclipsed by competing priorities. Reduced physical and psychological capability could then negatively impact motivation for some patients, leading to disengagement with the exercises. Indeed once patients resign themselves to total use of a feeding tube, it is likely that motivation diminishes. The caution to guard against tube dependency has been highlighted by others [43–45]. The importance of good multidisciplinary team working is essential as prophylactic feeding tubes may be necessary in some patients who are predicted to have severe dysphagia that may compromise completion of their chemo-radiation treatment [46, 47]. It is vital that patients are adequately counselled and monitored to prevent subtle shifts in motivation that may occur once a feeding tube is in place.

### Limitations and Future Directions

This study was undertaken on a small sample of patients, although a reasonably diverse group was achieved and a method for data saturation was specified. As with most qualitative studies, our findings may be context based, and therefore not widely generalized. However, we were not looking to find generalizable results, but rather to capture a range of patient views that may need addressing in future interventions. We have also provided a detailed methodology and encourage repeat studies in different contexts. While researcher subjectivity is a frequent concern in qualitative analysis, the availability of a codebook and the high percentage agreement obtained with a second independent coder suggest that the concepts have credence beyond the sole analysis and interpretation of the lead researcher/interviewer.

Further qualitative studies on barriers and facilitators to swallowing exercise adherence will be useful to expand upon this work. Recognizing that patient adherence is important to the success of interventions, future work is necessary to address how adherence is operationalized as a concept and how best to measure this in empirical studies. Other researchers have pointed out that adherence is sometimes reported on a continuum, and other times as a dichotomy with no clear consensus on how best to measure adherence to home-based swallowing exercises [20]. A recent study [48] concluded that HNC patients’ adherence to using electrical stimulation as a therapy to improve swallowing physiology had no impact on the efficacy of the treatment. However, we cannot extrapolate this finding to all forms of swallowing rehabilitation. Studies that aim to optimize adherence to swallowing exercises before and during treatment are still merited. Without this, we have little means of verifying whether swallowing exercises improve the swallowing function and QOL of patients with HNC.

## Conclusion

Patient *adherence* is one aspect of the complex intervention involved in swallowing rehabilitation after HNC. Researchers and clinicians working with dysphagic patients may wish to pro-actively consider ways of improving adherence when designing interventions [5]. This study described the use of a theory-based qualitative approach in examining what drives adherent/non-adherent exercise behaviours in patients with HNC. Insights gained by adopting this approach can help inform the development of new swallowing interventions for patients with HNC.

## Electronic supplementary material

Below is the link to the electronic supplementary material.

Supplementary material 1 (DOCX 114 kb)
