# Supplementary material for: Patient Experiences of Swallowing Exercises After Head and Neck Cancer: A Qualitative Study Examining Barriers and Facilitators Using Behaviour Change Theory
Source: Dysphagia. 2017 Apr 19;32(4):559–69. doi: 10.1007/s00455-017-9799-x (PMC5515965; doi:10.1007/s00455-017-9799-x)
Supplement: Supplementary file 1 — Supplementary material 1 (DOCX 114 kb) [file 455_2017_9799_MOESM1_ESM.docx]

Interview prompt questions based on the Theoretical Domains Framework.

*adapted from Michie, Atkins and West, 2014

| **Domain (definition)** | **Theoretical constructs represented within each domain** | **Interview prompt questions*** |
| --- | --- | --- |
| **Knowledge**  (An awareness of the existence of something) | Knowledge (including knowledge of condition /scientific rationale); Procedural knowledge; Knowledge of task environment | Can you tell me about how you got on with eating and drinking at the time of your treatment?  Can you tell me about anything you did or were advised to do to help with eating and drinking?  Can you tell me a bit about how/what happened when you were first given the exercises? |
| **Skills**  (An ability or proficiency acquired through practice) | Skills; Skills development; Competence; Ability; Interpersonal skills; Practice; Skill assessment | Were you able to perform the swallowing exercises?  How did you find the swallowing exercises? |
| **Memory, attention and decision processes**  (The ability to retain information, focus selectively on aspects of the environment and choose between alternatives) | Memory; Attention; Attention control; Decision making; Cognitive overload / tiredness | Did you feel that you would be able to carry out the exercises regularly as advised? |
| **Behavioural regulation**  (Anything aimed at managing or changing  objectively observed or measured actions) | Professional identity; Professional role; Social identity; Identity; Professional boundaries; Professional confidence; Group identity; Leadership; Organisational commitment | Did you have a way of monitoring whether you did the exercises regularly? |
| **Social/professional role and identity**  (A coherent set of behaviours and displayed personal qualities of an individual in a social or work setting) | Professional identity; Professional role; Social identity; Identity; Professional boundaries; Professional confidence; Group identity; Leadership; Organisational commitment |   Not applicable |
| **Beliefs about capabilities**  (Acceptance of the truth, reality, or validity about an ability, talent, or facility that a person can put to constructive use) | Self-confidence; Perceived competence; Self-efficacy; Perceived behavioural control; Beliefs; Self-esteem; Empowerment; Professional confidence | Were you confident that you were able to do the exercises correctly? |
| **Optimism**  (The confidence that things will happen for the best or that desired goals will be attained) | Optimism; Pessimism; Unrealistic optimism; Identity | What did you feel the exercises were doing for you? |
| **Beliefs about consequences**  (Acceptance of the truth, reality, or validity about outcomes of a behaviour in a given situation) | Beliefs; Outcome expectancies; Characteristics of outcome expectancies; Anticipated regret; Consequents | Did you feel that it was important to do the exercises? |
| **Intentions**  (A conscious decision to perform a behaviour/ act in a certain way) | Stability of intentions; Stages of change model; Transtheoretical model and stages of change | How did you decide whether or not to do the exercises? |
| **Goals**  (Mental representations of outcomes or end states that an individual wants to achieve) | Goals (distal / proximal) ; Goal priority; Goal / target setting; Goals (autonomous / controlled); Action planning; Implementation intention | Tell me about how much, and how often you did the exercises?  Were there days when you did not do the exercises – why was that?  Were there days when you did not feel like doing the exercises but did them anyway – what do you think made you do them? |
| **Reinforcement**  (Increasing the probability of a response by arranging a dependent relationship, or contingency, between the response and a given stimulus) | Rewards (proximal / distal, valued / not valued, probable / improbable); Incentives; Punishment; Consequents; Reinforcement; Contingencies; Sanctions | Was there anything that spurred you to do the exercises? |
| **Emotion**  (A complex reaction pattern, involving experiential, behavioural, and physiological elements, by which the individual attempts to deal with a personally significant matter or event) | Fear; Anxiety; Affect; Stress; Depression; Positive / negative affect; Burn-out | How did it make you feel having to do swallowing exercises? |
| **Environmental context and resources**  (Any circumstance of a person’s situation or environment that discourages or encourages the development of skills and abilities, independence, social competence, and adaptive behaviour) | Environmental stressors ; Resources / material resources ; Organisational culture /climate ; Salient events / critical incidents; Person x environment interaction; Barriers and facilitators | Can you tell me more about when and where you did the exercises? |
| **Social influences**  (Those interpersonal processes that can cause individuals to change their thoughts, feelings, or behaviours) | Social pressure; Social norms; Group conformity; Social comparisons; Group norms; Social support; Power; Intergroup conflict; Alienation; Group identity; Modelling |   Were other people around/involved when you were doing your exercises – How did they respond? |
